# The molecular weight of hyaluronic acid influences metabolic activity and osteogenic differentiation of periodontal ligament cells

**DOI:** 10.1007/s00784-023-05202-z

**Published:** 2023-08-17

**Authors:** Iris Frasheri, Nikoletta Dimitra Tsakiridou, Reinhard Hickel, Matthias Folwaczny

**Affiliations:** Department of Conservative Dentistry and Periodontology, University Hospital, Ludwig-Maximilians-University, LMU Munich, Goethestr. 70, 80336 Munich, Germany

**Keywords:** Periodontal ligament, Osteogenesis, Osteogenic differentiation, Hyaluronan, Molecular weight

## Abstract

**Objective:**

While HA is present naturally in periodontal tissues, its molecular weight can vary widely in vivo. The objective of this study was to directly compare the biological reactions of periodontal ligament cells to four distinct molecular weights of hyaluronic acid (HA).

**Materials and methods:**

Immortalized human periodontal ligament cells (PDL-hTERT) were cultured for 21 days in culture medium alone (control) or enriched with osteogenic supplements (OS group). Other 4 experimental groups were cultured in OS medium with the addition of HA with different molecular weights (HMW, MMW, LMW, and ULMW). The cell morphology was examined daily. WST1 assays were performed to evaluate metabolic activity. Von Kossa staining and calcium deposition assay were used to analyze osteogenic differentiation and mineralization.

**Results:**

Cell morphology remained unaltered in all groups. Cells stimulated with OS alone or with the addition of hyaluronan showed all the typical microscopic appearance of osteogenic differentiation. Metabolic activity increased in all groups over time. Hyaluronan stimulated greater metabolic activity than the control group, with LMW HA and MMW HA showing the most significant increase. All groups showed mineral deposits and calcium deposition after 21 days of stimulation.

**Conclusion:**

Our results suggest that hyaluronan can promote metabolic activity and mineralization of PDL-hTERT cells, with LMW HA being the most effective.

**Clinical relevance:**

These results shed light on how the various molecular weight fractions of HA promote tissue regeneration and repair, as well as help to identify an optimal molecular weight range for this application in periodontal tissues.

## Introduction

Periodontitis is a chronic inflammatory disease that affects the supporting structures of teeth, including the periodontal ligament and alveolar bone [1]. The extracellular matrix of these tissues is primarily composed of a network of glycosaminoglycans (GAGs) [2], among which hyaluronic acid (HA) plays a crucial role. HA is a linear polymer of repeating disaccharide units of glucuronic acid and N-acetylglucosamine [3], and its molecular weight (MW) can vary widely in vivo, ranging from ultra-low (ULMW) to high (HMW) molecular weight [4].

This non-sulfated glycosaminoglycan HA in its native form is present as a long linear polysaccharide having a high-molecular weight >1000kDa (HMW HA) [5, 6]. Three hyaluronan synthase enzymes are responsible for the synthesis of HA and its release of the protein into the intercellular space HAS 1, 2 and 3 [7].

HA has been found to be present naturally in periodontal tissues, gingival connective tissues as well as gingival epithelium [8, 9] produced/synthesized by the hyaluronan synthases at the plasma membrane [10, 11] of various cells in the periodontal area including epithelial rest of Malassez cells [12], and periodontal ligament cells, with fibroblasts being the primary cell type responsible for the synthesis of HA in the periodontal tissues [13]. Particularly, Utsunomiya et al. found HA to be more localized at edematous/mixoid areas of the periodontal ligament than in compact fibrous areas [9].

The content of HA in human gingival epithelium has been calculated to be circa 5.58 μg/g dry wt., while in human gingival connective tissue circa 126.4 μg/g dry wt. [8].

Several studies have suggested an important role for hyaluronan in maintaining periodontal tissue homeostasis [14]. Moreover, HA has been shown to regulate inflammatory responses by modulating the activity of immune cells, particularly neutrophil functions [15, 16], and regulating cytokine expression [17]. Furthermore, it has been proposed that the presence of HA in periodontal tissues could play a key role in regulating cell proliferation, differentiation, and wound healing [18].

In case of inflammation, hyaluronidases, the activity of reactive oxygen and nitrogen species, etc. induce the HA into smaller fragments of different MW [19, 20]. In fact, while in healthy gingiva there is a predominance of native HA, it has been evidenced that in gingiva of patients with periodontal disease there is a shift towards low-molecular size glycosaminoglycans [21].

The amount of hyaluronan, specifically the low-molecular weight HA (LMW HA) present in the gingival crevicular fluid (GCF) has been seen to be higher in patients affected by periodontitis, even after periodontal therapy [22].

These apparently different involvements of HA, support the suggestion of Wu et al, that studying HA in knee osteoarthritis, suggested that HA of different MW present peculiar features and they should be not considered as a single group [23].

Even having evidenced the presence of fractions with a different molecular weight HA, the present available literature is scarce in comparing their effects in the periodontal tissues.

Understanding the effects of different molecular weight fractions of HA on PDL cells is important for elucidating the mechanisms involved in periodontal tissue maintenance and repair. By investigating these effects, we can also gain insight into the optimal molecular weight range for promoting tissue regeneration and repair.

Thus, the aim of this study was to directly compare and better understand the biological reactions of PDL-hTERT cells to four distinct molecular weights of hyaluronan.

## Materials and methods

### Cell culture

Human immortalized periodontal ligament cells (PDL-hTERT) were established as previously described [24]. PDL-hTERT cells were maintained at 5% CO2 and 37°C in Dulbecco's Modified Eagle Medium (DMEM, high glucose, +Glutamine, +Pyruvate) enriched with 10% fetal bone serum, 1% Pen-Strep solution and 2mM L-glutamine (all Sigma Aldrich, Munich, Germany). Medium change was performed twice a week.

### Stimulating conditions and cell morphology

A control group of PDL-hTERTs was cultured in DMEM enriched as described above, without any hyaluronan supplements. Another group was constituted of PDL-hTERTs cultured in osteogenic differentiation medium (OS), which was DMEM with osteogenic supplement: 10% FBS, 1% Pen-Strep, 100nM Dexamethasone, 10mM β-glycerol phosphate and 50μM L-ascorbic acid (Sigma Aldrich, Munich, Deutschland).

Hyaluronan (HA) with different molecular weights was purchased from R&D Systems, Minneapolis, MN, USA. The four groups further used for the experiments were as follows: High Molecular Weight Hyaluronan (HMW), Medium Molecular Weight Hyaluronan (MMW), Low Molecular Weight Hyaluronan (LMW), and Ultra Low Molecular Weight Hyaluronan (ULMW). HMW hyaluronan is provided by the company in a molecular mass range of >950kDa. The specific lot used had a mass of 1510kDa. MMW hyaluronan presents a molecular mass range of 75–350kDa. The lot used had a mass of 229kDa. LMW hyaluronan has a molecular mass range of 15–40kDa. The specific lot used had a mass of 37kDa. ULMW hyaluronan is in a molecular mass range of 4–8kDa. The specific lot used had a mass of 4.6kDa. HA was reconstituted as per manufacturer´s indications in phosphate buffered saline and sterilized by filtration with 0.2-μm syringe filters (VWR International, Radonor, USA). The stimulating concentration of 100μg/ml was further used in the experimental groups, and it was determined based on previous studies [25, 26]. PDL-hTERTs between the 27^th^ and the 29^th^ passage were used. PDL-hTERT were cultured in 1 ml medium, at 2x 10^4^ cells/well in 12-well plates for 3 weeks.

The cell morphology was examined daily, and photographs were taken using a phase contrast microscope (Axiovert, Carl Zeiss, Jena, Germany). For each studied group, the experiments were performed in triplicates and repeated three times.

### Metabolic activity

Water-soluble tetrazolium-1 (WST-1) assays were performed. For this assay, PDL-hTERTs were seeded in 24-well plates (1x10^4^ cells/well) and cultured for 7, 14, or 21 days, exposed to the above-mentioned HA solutions (HMW, MMW, LMW and ULMW). The groups with medium alone and OS medium were also cultured under the same conditions. Medium change with 0.5 ml per well was performed every 2 days. At each experiment day, WST-1 (Roche Diagnostics GmbH, Mannheim, Germany) was added according to manufacturer’s protocols in a 1:10 final dilution, incubated for 1 hour (5% CO_2_ and 37°C). The medium of each well was transferred into two wells of a 96-well plate, with a volume of 100 μl per well. The optical density of the samples was measured through a multi-detection microplate reader (TECAN, Infinite M200, Männedorf, Switzerland). Data analyses were performed with Magellan™ Software (TECAN). Absorbance readings were recorded at 450nm, with a wavelength correction at 650nm. The experiments were performed in triplicates and repeated three times.

### Von Kossa

Von Kossa staining was used to analyze mineralization in cell cultures in the six study groups.

PDL-hTERT were seeded at 2x 10^4^ cells/well in 12-well plates and cultured for 21 days in the same media as described above. The respective media were replaced twice a week.

The cells were afterwards fixed for 20 min in methanol at -20°C. The standard protocol was used for the staining with silver nitrate (Merck KGaA, Darmstadt, Germany), pyrogallol acid solution (University Pharmacy, Munich, Germany), sodium hydroxide solution (Merck KGaA, Darmstadt, Germany) and May-Grünwald solution (Merck KGaA, Darmstadt, Germany). Afterwards, pictures were taken in standardized conditions for all groups.

### Calcium deposition assay

PDL-hTERTs in the 28^th^ passage were plated at a concentration of 2x 10^4^ cells/well in 12-well plates and divided into 6 groups as above: control; +OS; +OS+ HMW, +OS+ MMW, +OS+ LMW and +OS+ ULMW. The cells were stimulated for 21 days and then harvested with QuantiChrom^TM^ Calcium Assay Kit (Bioassay Systems, Basel, Switzerland) following manufacturer´s protocol. The calcium quantification was done with a microplate reader (TECAN, Infinite M200, Männedorf, Switzerland). The measurements were performed at 612 nm and the calculations of calcium deposition in mg/dl were done establishing a standard curve as per manufacturer´s indications.

### Statistics

Statistical analyses were performed using SPSS software (Version 26.0, SPSS Statistics, IBM, Chicago, IL, USA). Within each group, data were tested for normality with the Kolmogorov-Smirnov and Shapiro-Wilk tests. Homogeneity of variances between groups was tested with Levene test. Since data were not normally distributed, differences between study groups in calcium deposition for each time point were calculated with the Mann-Whitney *U* test and with Games-Howell test for WST-1. The *p*-value threshold was set at 0.05. If not otherwise indicated, all data are given as mean ± standard deviation (±SD).

## Results

### Cell morphology

**Fig. 1:**
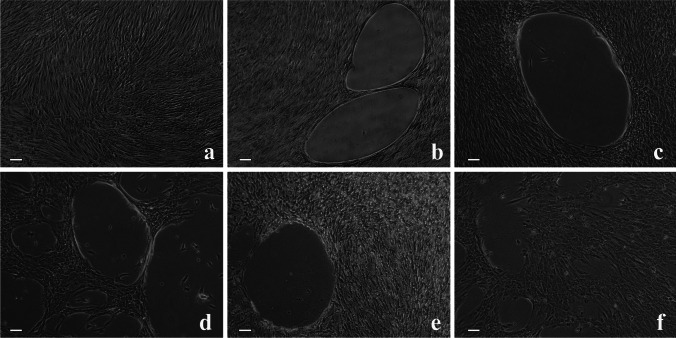
Microscopy of PDL-hTERTs after 14 days. Unstimulated PDL-hTERTs cultured in a pure culture medium served as controls (**a**). PDL-hTERTs were cultured in the presence of osteogenic medium (OS; **b**) or stimulated with osteogenic medium and different molecular weights (MW) hyaluronan: high MW (HMW; **c**), medium MW (MMW; **d**), low MW (LMW; **e**) or ultra-low MW (ULMW; **f**). Magnification ×5. The scale bars represent 50 μm

The single cell morphology remained unaltered in all groups, which presented an elongated spindle shaped form (Fig. 1). The cells grew in a monolayer with increased density until day 7. During the second week in culture, they formed a rolled aggregate with oval-shaped, cell-free areas, typical of osteogenic differentiation. This was observed in all groups stimulated with osteogenic medium, as well as in the ones with hyaluronan supplements but, as expected, not in the control. Hyaluronan did obviously not induce any morphological changes in PDL-hTERT cells irrespectively from the particular molecular weight (Fig. 1).

### Metabolic activity

**Fig. 2:**
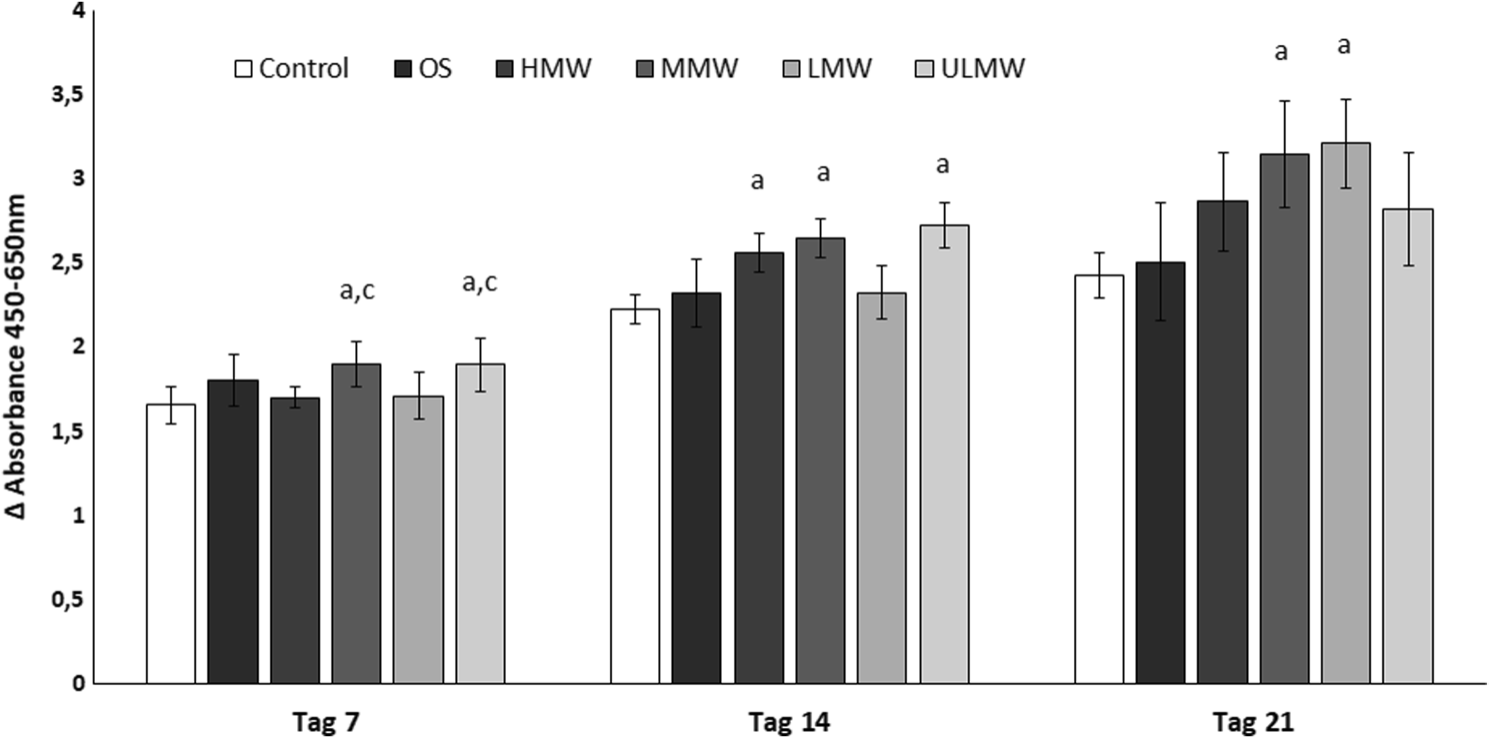
WST-1 cell proliferation assay at day 7, 14 and 21 of PDL-hTERT cells in the presence of osteogenic medium (OS) or stimulated with osteogenic medium and different molecular weights (MW) hyaluronan: high MW (HMW), medium MW (MMW), low MW (LMW) or ultra-low MW (ULMW). Unstimulated PDL-hTERTs cultured in a pure culture medium served as controls. Results are presented as mean values (± standard deviation). Differences between groups have been considered significant for *p* < 0.05 and marked above the respective column with the letters “a” material vs. control, “b” material vs OS, “c” vs HMW, “d” vs. MMW, “e” vs. LMW, “f” vs. ULMW

Considering the changes within the single study groups overtime, in all six groups, the cells´ activity increased with time, so that the highest value was measured for all groups on the 21st day. The osteogenic and the control groups increased equally within the observed time intervals and reached comparable values (Fig. 2). Accordingly, there was no significant difference between the two groups (*p*> 0.05). The maximum increase in cell activity within the observed 21 days was seen in the cells stimulated with LMW and MMW. While the cells stimulated with MMW showed a higher difference in their metabolic activity from day 7 to day 14, this was more the case between day 14 and 21 for the cells stimulated with LMW. Moreover, it can be observed that the groups stimulated with HMW, MMW and ULMW hyaluronan presented at day 14 significantly higher metabolic activity than the control group.

A significant difference (*p*<0.05) between HMW group and ULMW can also be observed at day 7, which, however, could no longer be detected at day 14. At day 21, all groups stimulated with hyaluronic acid presented higher values than the control, but also higher than the OS group, but this reached significance just for LMW and MMW in the comparison with the control.

### Mineralization

**Fig. 3:**
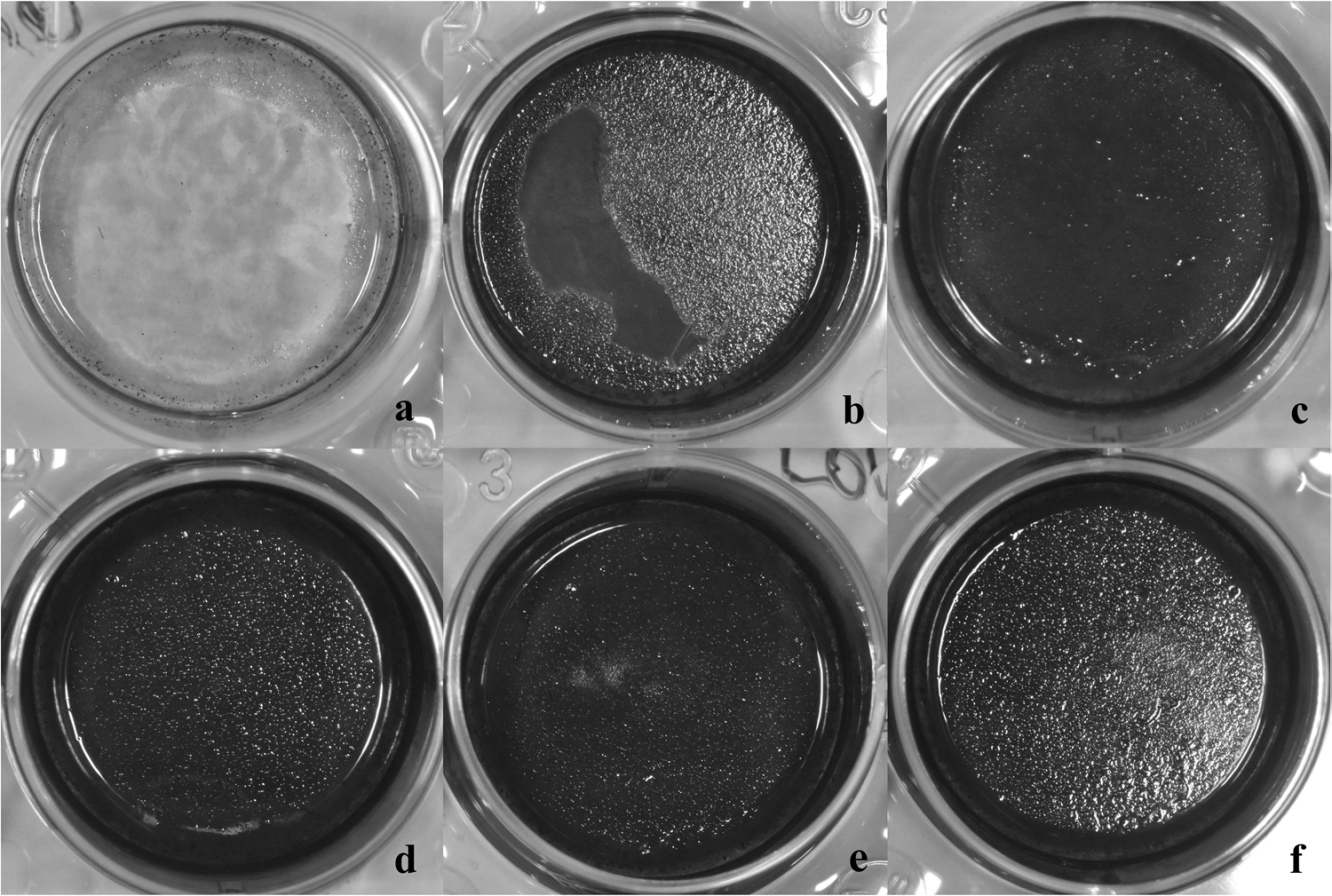
Von Kossa staining at day 21 of PDL-hTERT cells unstimulated (control; **a**) or in the presence of osteogenic medium (OS; **b**) or stimulated with osteogenic medium and different molecular weights (MW) hyaluronan: high MW (HMW; **c**), medium MW (MMW; **d**), low MW (LMW; **e**) or ultra-low MW (ULMW; **f**)

We next assessed the capacity of PDL-hTERT cells stimulated with different molecular weight hyaluronan to differentiate into osteoblasts capable of forming a mineralized extracellular matrix. Mineral deposits by PDL-hTERTs were identified by von Kossa staining technique in all groups after 21 days of stimulation. Photographs of representative cultures are shown in Fig. 3.

**Fig. 4:**
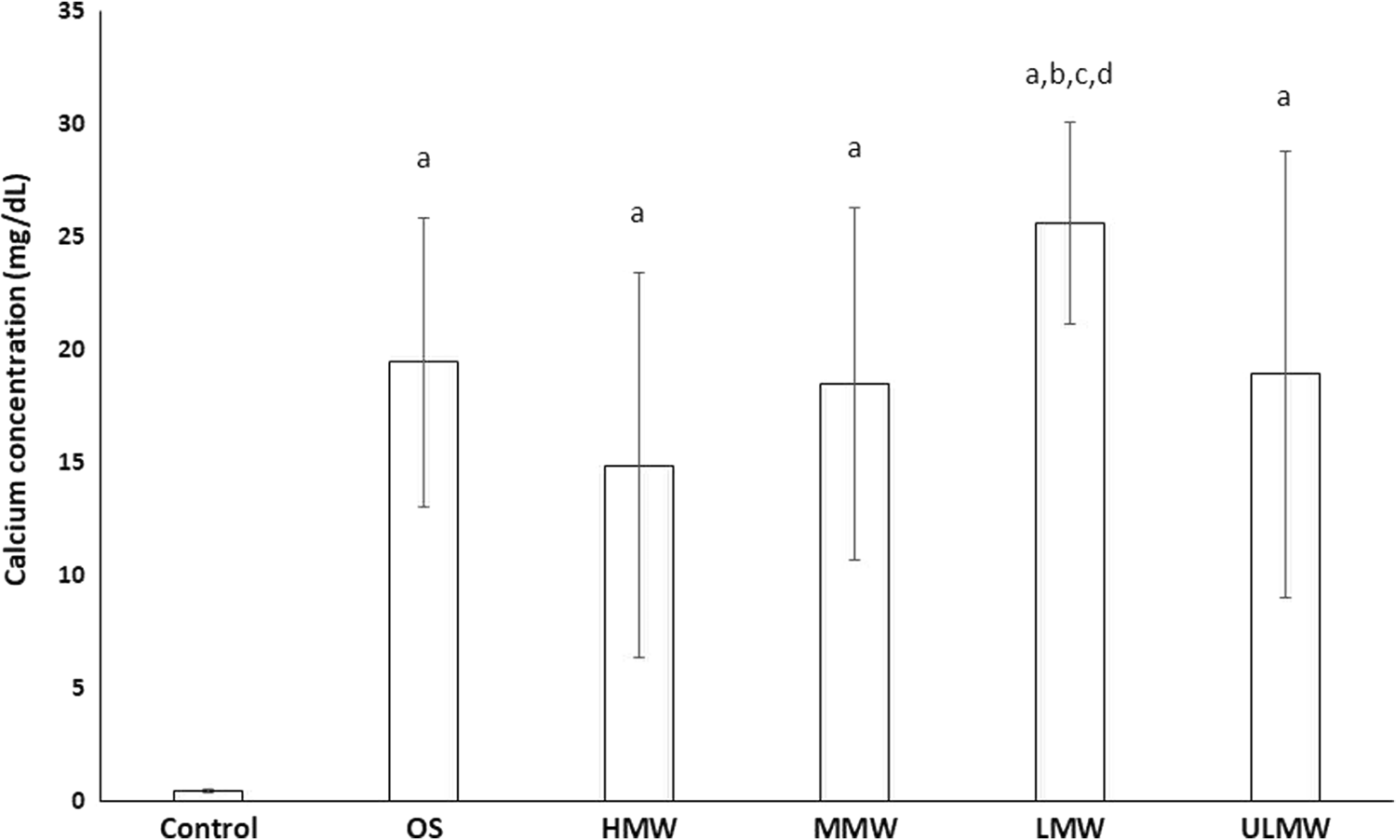
Calcium deposition assay at day 21 of PDL-hTERT cells unstimulated (control; **a**) or in the presence of osteogenic medium (OS; **b**) or stimulated with osteogenic medium and different molecular weights (MW) hyaluronan: high MW (HMW; **c**), medium MW (MMW; **d**), low MW (LMW; **e**) or ultra-low MW (ULMW; **f**). Results are presented as mean values (± standard deviation). Differences between groups have been considered significant for *p* < 0.05 and marked above the respective column with the letters “a” material vs. control, “b” material vs OS, “c” vs HMW, “d” vs. MMW, “e” vs. LMW, “f” vs. ULMW

The mineralization was also quantified in parallel experiments by calcium deposition assay (Fig. 4). Spectrophotometric quantification confirmed these observations, revealing a significantly increased absorbance at 450 nm and calculated calcium deposition, for the cells stimulated with LMW. The osteogenic parameters investigated were decreased in the group stimulated with HMW, but this difference did not reach significance.

## Discussion

The current study aimed to determine the influence of fragments of hyaluronic acid with varying molecular weights on periodontal ligament cells. This influence was evaluated first on the morphological, phenotypical expression of PDL-hTERTs, and then on the capacity of these cells to differentiate into osteoblasts, as described by Docheva et al. [24]. The PDL-hTERTs were specifically chosen due to their multipotent properties.

Furthermore, these fibroblasts are the main cells occurring in the periodontal crevicular space, performing several functions in periodontal regeneration and homeostasis [27, 28]. Consequently, they represent an appropriate experimental model for investigations in the context of periodontal therapy after adequate stimulation. In order to provide a comprehensive overview, we used four different sizes of hyaluronic acid, all applied at the same concentration to allow better comparison.

Regarding microscopic appearance, osteogenically stimulated PDL-hTERTs as well as those additionally treated with any of the fragments of HA showed an ongoing osteogenic differentiation at day 14. Contrarily to the control, all treated groups exhibited oval-shaped, cell-free areas that are typical of the beginning of mineralization/osteogenic differentiation. No differences were seen to be induced in cell morphology by HA, compare to OS alone. This has also been demonstrated by Kawano et al. for HMW hyaluronic acid of more than 2000kDa [29]. However, in this study, no other hyaluronan fragments were tested.

Analyzing metabolic activity, we observed a significant increase in PDL-hTERTs proliferation treated with MMW HA, already at day 7, until the end of the observation period, after 21 days, which is greater than the control and any hyaluronan with different MW. Similar results were found for the proliferation rate of skin keratinocytes by Ghazi et al., who hypothesized that MMW HA is a more effective therapeutic approach than native HA for promoting wound healing [30]. In line with this, a clinical study showed that the topical use of HMW HA reduces cell proliferation in fibroblasts, epithelial cells, lymphocytes, etc. [31]. Also an in vitro study on dental pulp stem cells showed that LMW HA induces a proliferation rate that is two times higher than that of HMW HA.

A recent study conducted on hamster ovary cells demonstrated that the application of sodium hyaluronate with a molecular weight > 1300kDa [32] inhibits proliferation in a concentration-dependent manner [33], particularly for concentrations > 0.5mg/ml. In the current study, we observed lower metabolic activity in PDLhTERTs treated with HMW HA at day 7, although not reaching significance. However, at day 14, there was a significant increase in metabolic activity. Nevertheless, the anti-inflammatory functions of high molecular weight hyaluronate may be a key factor in its efficacy for periodontal applications, as suggested by previous research [6]. According to results obtained by Tanimoto et al., HA may contribute to regulating this proliferation of human PDL cells through a mechanism mediated by CD44 [34].

The effect of LMW HA has been analyzed in 3D culture models and it has been demonstrated to enhance osteogenesis and bone matrix formation [35]. This is consistent with our results, which showed a significantly higher deposition of calcium salts after 21 days of treatment with LMW HA.

In contrast, ULMW HA in our study appeared to have no effect on cell morphology or mineralization potential of PDL-hTERTs. However, it induced a greater metabolic activity in PDL-hTERTs at the beginning of their osteogenic differentiation, at day 7 and 14.

In a study by Ariyoshi et al. [36], HA with a molecular weight of less than 8kDa HA, comparable to our ULMW HA, seemed to also enhance bone resorption, upregulating osteoclast differentiation.

It is worth considering that the biological effects HA can be cell type or tissue specific, as suggested by Cowman et al. [4]. Therefore, HA fragments of varying sizes can assume different roles, as the levels of the receptors or of signaling proteins are heterogeneous in different areas [37]. Additionally, several studies use different concentrations as well as slightly different ranges to define the various molecular weight fractions of HA (in kDa), which can make comparisons challenging. From this point of view, our study has the advantage of directly comparing multiple fractions, thus providing a more comprehensive understanding of their effects. At last, another important point to consider in comparing the functional effects of hyaluronic acid is that different animal species present a different MW HA which is physiologically more present in a specific tissue.

A limitation of this study is that it is performed with immortalized PDL cells isolated from one donor and, therefore, might not be representative of a general population. Another limitation of our study is that being an in vitro study, it lacks the possible mutual influence between HA fragments and the surrounding tissues, as these interactions influence the growth microenvironment of the cells regulating the HA response [38].

In the light of our results, the molecular weight of the local HA could be used for its potential predictive value in amplifying periodontal regenerative healing. In terms of the current results particularly HA fragments with medium molecular weight are promising candidates to further delineate the regenerative potential of glycosaminoglycans, specifically HA.

Also, the concentration of HA fragments might influence the obtained cell effects. Thus, further studies might consider covering a wider range for HA concentrations. It might be also interesting to investigate combined effects of HA with different molecular weights, as a bio-synergistic impact of two distinct molecular weights of HA was also proposed by Rosaming et al. [39].

## Conclusions

Overall, our in vitro findings suggest that the molecular weight of HA may play a key role in regulating the mineralization of periodontal tissues, and further investigation is needed to improve the understanding of the underlying biological mechanisms involved.
